# Gamma-Glutamyltransferase Fractions in Human Plasma and Bile: Characteristic and Biogenesis

**DOI:** 10.1371/journal.pone.0088532

**Published:** 2014-02-12

**Authors:** Irene Fornaciari, Vanna Fierabracci, Alessandro Corti, Hassan Aziz Elawadi, Evelina Lorenzini, Michele Emdin, Aldo Paolicchi, Maria Franzini

**Affiliations:** 1 Department of Biology, University of Pisa, Pisa, Italy; 2 Department of Translational Research and New Technologies in Medicine and Surgery, University of Pisa, Pisa, Italy; 3 Cardiology and Cardiovascular Medicine Division, Fondazione Toscana G. Monasterio – CNR, Pisa, Italy; 4 Life Science Institute, Scuola Superiore Sant'Anna, Pisa, Italy; Institute of Infection and Global Health, United Kingdom

## Abstract

Total plasma gamma-glutamyltransferase (GGT) activity is a sensitive, non-specific marker of liver dysfunction. Four GGT fractions (b-, m-, s-, f-GGT) were described in plasma and their differential specificity in the diagnosis of liver diseases was suggested. Nevertheless fractional GGT properties have not been investigated yet. The aim of this study was to characterize the molecular nature of fractional GGT in both human plasma and bile.

Plasma was obtained from healthy volunteers; whereas bile was collected from patients undergoing liver transplantation. Molecular weight (MW), density, distribution by centrifugal sedimentation and sensitivity to both detergent (deoxycholic acid) and protease (papain) were evaluated. A partial purification of b-GGT was obtained by ultracentrifugation.

Plasma b-GGT fraction showed a MW of 2000 kDa and a density between 1.063–1.210 g/ml. Detergent converted b-GGT into s-GGT, whereas papain alone did not produce any effect. Plasma m-GGT and s-GGT showed a MW of 1,000 and 200 kDa, and densities between 1.006-1.063 g/ml and 1.063–1.210 g/ml respectively. Both fractions were unaffected by deoxycholic acid, while GGT activity was recovered into f-GGT peak after papain treatment. Plasma f-GGT showed a MW of 70 kDa and a density higher than 1.21 g/ml. We identified only two chromatographic peaks, in bile, showing similar characteristics as plasma b- and f-GGT fractions.

These evidences, together with centrifugal sedimentation properties and immunogold electronic microscopy data, indicate that b-GGT is constituted of membrane microvesicles in both bile and plasma, m-GGT and s-GGT might be constituted of bile-acid micelles, while f-GGT represents the free-soluble form of the enzyme.

## Introduction

γ-Glutamyltransferase (GGT, EC 2.3.2.2) is a dimeric glycoprotein consisting of a heavy and a light subunit linked by non-covalent bonds. In the N-terminal portion of the heavy subunit a hydrophobic domain allows the enzyme to be anchored to cell plasma membrane, with both subunits exposed in the extracellular environment [1]. GGT catalytic site is localized in the light subunit and acts on extracellular substrates, such as glutathione (GSH) [2], leukotriene C4, nitrosoglutathione and other GSH-conjugates [3], [4]. GGT is present in the plasma membrane of virtually all cells, but it is mainly localized in the epithelial tissues with secretory or absorptive functions. In adult liver, GGT is localized in the biliary pole of hepatocytes and in cholangiocytes and hence secreted in bile [3], [5]. Plasma GGT is supposed to origin from liver [6], [7] and early studies demonstrated that it is associated with several carriers having different molecular weights, densities and charges [8], [9]. Nevertheless, the mechanism of release, the structures and the clinical significance of such carriers have not been completely characterized.

As regard clinical applications, plasma GGT values over the upper reference limit (men: 54 U/L, women: 34 U/L) [10] are found in different types of hepatobiliary diseases, regardless of the aetiology and alcohol abuse [3]. Studies conducted over the past 10 years have shown that plasma GGT values in the upper part of the reference range (men>28 U/L; women>19 U/L) [11] are positively associated with a higher risk of cardiovascular events [11]–[13], onset of type II diabetes [14], [15] and metabolic syndrome [16], independently of liver diseases and alcohol consumption. Nevertheless, the currently used laboratory GGT assay does not allow the discrimination among the different causes of increased plasma GGT levels, thus reducing its clinical value and specificity as a sensitive disease biomarker.

Recently, we developed a sensitive and reproducible method that allowed us to identify four GGT fractions in plasma of all healthy subjects, named big-GGT (b-GGT), medium-GGT (m-GGT), small-GGT (s-GGT) and free-GGT (f-GGT), with molecular weight (MW) corresponding to 2000, 1000, 250, and 70 kDa, respectively [17]. While f-GGT is the most abundant fraction in healthy adults [18], the three high MW fractions (b-, m- and s-GGT) are increased in liver diseases [19]. The first study on diagnostic specificity of the four GGT fractions showed that the latter are present also in patients with non-alcoholic fatty liver diseases (NAFLD), chronic viral hepatitis C [19] or alcoholic liver disease [20], but in different quantities. Thus the different GGT fractions patterns, despite similar total GGT values, allowed the differential diagnosis [19], [20].

The aim of this study was to evaluate the characteristics of plasma GGT carriers and to show if the enzyme is entire or devoid of the N-terminal hydrophobic peptide which is thought to be responsible of the interaction between GGT and the carriers. Physical properties (*i.e.* dimension and density), as well as sensitivity of each fraction to detergent (deoxycholic acid) and protease (papain) treatment were evaluated. Bile GGT, possibly contributing to circulating GGT, was also studied.

## Materials and Methods

### Ethics Statement

The Human Ethics Committee of the University Hospital of Pisa approved the study; all subjects gave a written informed consent.

### Plasma and bile samples

Aliquots (1–2 ml) from anonymous plasma EDTA samples used for routine analysis were provided from the Department of Medical Laboratory of Fondazione Toscana G. Monasterio.

Different samples were pooled to provide suitable volumes for a more accurate lipoprotein separation and recovery and to obtain sufficiently concentrated exosome pellets to be analyzed by immunogold.

Anonymous bile samples (3–8 ml) were obtained from hospitalized patients undergoing allogeneic liver transplantation at the Hepatobiliary Surgery and Liver Transplant Unit of the University Hospital of Pisa. Samples were collected from Kehr's T tube placed in the common bile duct during the implant of liver allograft.

### Separation and quantification of GGT fractions

Separation and quantification of GGT fractions were performed on plasma (injection volume V_i_: 0.02 ml), plasma lipoproteins (V_i_: 0.05 ml), bile (V_i_: 0.02 ml), microparticles (V_i_: 0.05 ml) and exosomes (V_i_: 0.05 ml) samples, these latter obtained from both plasma and bile.

All samples were centrifuged (3,000 g, 3 min, 4°C) and supernatants were filtered using a 0.45 µm PVDF filter. Analysis of total and fractional GGT was performed as previously described [17]. Briefly, a fast protein liquid chromatography (FPLC) system (AKTA purifier; GE Healthcare Europe, Milan, Italy) equipped with a gel-filtration column (Superose 6 HR 10/300 GL; GE Healthcare Europe) and a fluorescence detector (Jasco FP-2020; Jasco Europe, Lecco, Italy) was used. GGT activity was quantified by post-column injection of the fluorescent substrate for GGT, gamma-glutamyl-7-amido-4-methylcoumarin (gGluAMC, Nova Chimica, Milano, Italia). Total area, between 10 and 25 ml elution volumes, and fractional GGT areas were calculated by a MatLab program (Version 7 MathWorks, Inc.) to resolve overlapping peaks. The reaction was calibrated analysing plasma samples with known total GGT activity (standards); the slope of the calibration curve was used to convert total and fractional GGT areas in activity values expressed as U/L.

### Determination of total cholesterol

The elution profile of total cholesterol in plasma samples (Vi: 0.05 ml) and lipoprotein preparations (Vi: 0.05 ml) was obtained by using the same chromatographic procedure described for fractional GGT analysis. A suitable, commercially available reagent for total cholesterol determination (Giesse Diagnostic, Rome, Italy) was used as post-injected reagent. Reaction product was detected by recording the absorbance at 510 nm.

### Plasma lipoprotein separation

Plasma lipoproteins were separated by ultracentrifugation on a NaBr discontinuous density gradient [21] by using a Beckman ultracentrifuge with a fixed angle rotor (type 40). Four independent plasma pools (5 ml) – each obtained mixing five plasma aliquots – were examined. This method allowed us to separate very low-density lipoprotein (VLDL, d<1.006 g/ml), low-density lipoprotein (LDL, 1.006<d<1.063 g/ml) and high-density lipoprotein (HDL, 1.063<d<1.21 g/ml). As regard VLDL isolation, a 11.42 mg/ml NaCl solution (1 ml; d = 1.006 g/ml) was added to plasma pools (5 ml), then they were subjected to ultracentrifugal separation (100,000 g, 24 h, 20°C) and the VLDL containing supernatants (0.5 ml) were collected.

LDL isolation was obtained from VLDL-free plasma samples by adding a 11.42 mg/ml NaCl solution (0.5 ml; d = 1.006 g/ml) and a 434,80 mg/ml NaBr solution (1 ml; d = 1.3199). Samples, presenting with a 1.063 g/ml final density, were subjected to ultracentrifugal separation (100,000 g, 24 h, 20°C) and the LDL containing supernatants (0.85 ml) were collected. Finally, HDL isolation was obtained from VLDL- and LDL-free plasma samples by adding a 85.01 mg/ml NaBr solution (0.850 ml; d = 1.063 g/ml) and a 644.97 mg/ml NaBr solution (3 ml; d = 1.4705 g/ml). Samples, presenting with a 1.21 g/ml final density, were subjected to ultracentrifugal separation (100,000 g, 24 h, 20°C) and the HDL containing supernatants (0.79 ml) were collected.

### Plasma and bile treatment with papain and deoxycholic acid (DOC)

The cysteine protease papain (Sigma-Aldrich, Milan, Italy) was pre-incubated with a 25 mM acidic solution of cysteine (Cys-HCl; 3 min, RT) to achieve its full activation. Subsequently, plasma and bile samples were incubated with activated papain (20 U) for 24 h, RT under vigorous agitation.

As regard detergent treatment, plasma and bile samples (0.2 ml) were incubated with DOC (10 g/l, final concentration) for 1 h, RT under vigorous agitation. This experiment was repeated on four independent plasma or bile samples.

### Isolation of plasma and bile microparticles and exosomes

Isolation of microparticles and exosomes from plasma [22], [23] and bile [24] was performed by a differential centrifugation procedure. Briefly, samples (1 ml) were diluted to 2 ml with Dulbecco's phosphate buffered saline (PBS) and centrifuged at 2,000 g for 10 min at 4°C. Supernatants were then transferred into ultracentrifuge tubes, further diluted to 7 ml with cold PBS and centrifuged at 10,000 g for 45 min at 4°C to remove large debris. Again, supernatants were collected, transferred into ultracentrifuge tubes and centrifuged at 100,000 g for 2 h. Finally, the microvesicles-free supernatants were collected and the exosomes containing pellets were suspended in 0.2 ml of PBS. This experiment was repeated on five independent plasma samples and four independent bile samples.

### Exosomes preparation for electron microscopy

A plasma pool (100 ml, obtained mixing fifty plasma aliquots) was diluted with an equal volume of PBS and firstly centrifuged at 2,000 g for 15 min at 15°C. The obtained supernatant was collected and centrifuged at 15,000 g for 45 min at 15°C. Again, the post-centrifugal supernatant was recovered, filtered through a 0.22 µm filter to remove lipid aggregates and centrifuged at 100,000 g for 2 h at 15°C. Finally, the exosomes containing pellet was washed with PBS (100,000 g for 90 min at 15°C) and suspended in 40 ml of 0.15 M NaCl. Such exosomes suspension was then layered on 4 ml of a 30% w/v sucrose solution prepared in NaCl 0.15 M, and centrifuged at 100,000 g for 90 min at 4°C. A 4 ml aliquot was recovered from the bottom of the tube, diluted with 15 volumes of 0.15 M NaCl and centrifuged again at 100,000 g for 90 min at 4°C. The final pellet was then fixed in 1% paraformaldehyde.

A bile pool (120 ml, obtained mixing twenty bile samples) was diluted with an equal volume of PBS and centrifuged at 2,000 g for 30 min at 4°C. The obtained supernatant was collected and further centrifuged at 15,000 g for 30 min at 4°C. Again, the resulting supernatant was recovered and ultracentrifuged at 100,000 g for 2 h at 4°C. The final pellet was washed with PBS (100,000 g for 90 min at 4°C) and fixed in 2% paraformaldehyde.

### Immunogold electron microscopy and negative staining

Exosomes prepared from both plasma and bile (5 µl) were placed on an electrically charged (by glow discharge) copper grid covered with a collodion/amyl acetate membrane. Free aldehyde groups were saturated with a 50 mM NH_4_Cl solution in PBS (5 min, RT). Samples were incubated with a blocking solution (10% fetal bovine serum (FCS), 20 min, RT), then with a rabbit polyclonal primary antibody (1∶250 in 5% FCS, 1 h, RT) directed against the C-terminal 20 amino acids of human GGT heavy chain, and prepared as described [5]. The antigen-antibody reaction was revealed by using a secondary 15 nm gold particle-conjugated anti-rabbit IgG antibody (Emsdiasum; 1∶40 in 0.5% FCS, 60 min, RT). All samples were fixed with a 0.5% glutaraldehyde, 0.15 M NaCl solution (10 min, RT) and negatively stained with uranyl acetate. Finally, samples were examined under a transmission electron microscope (Philips 410).

## Results

### Plasma GGT fractions: dimension and density

**Figure 1:**
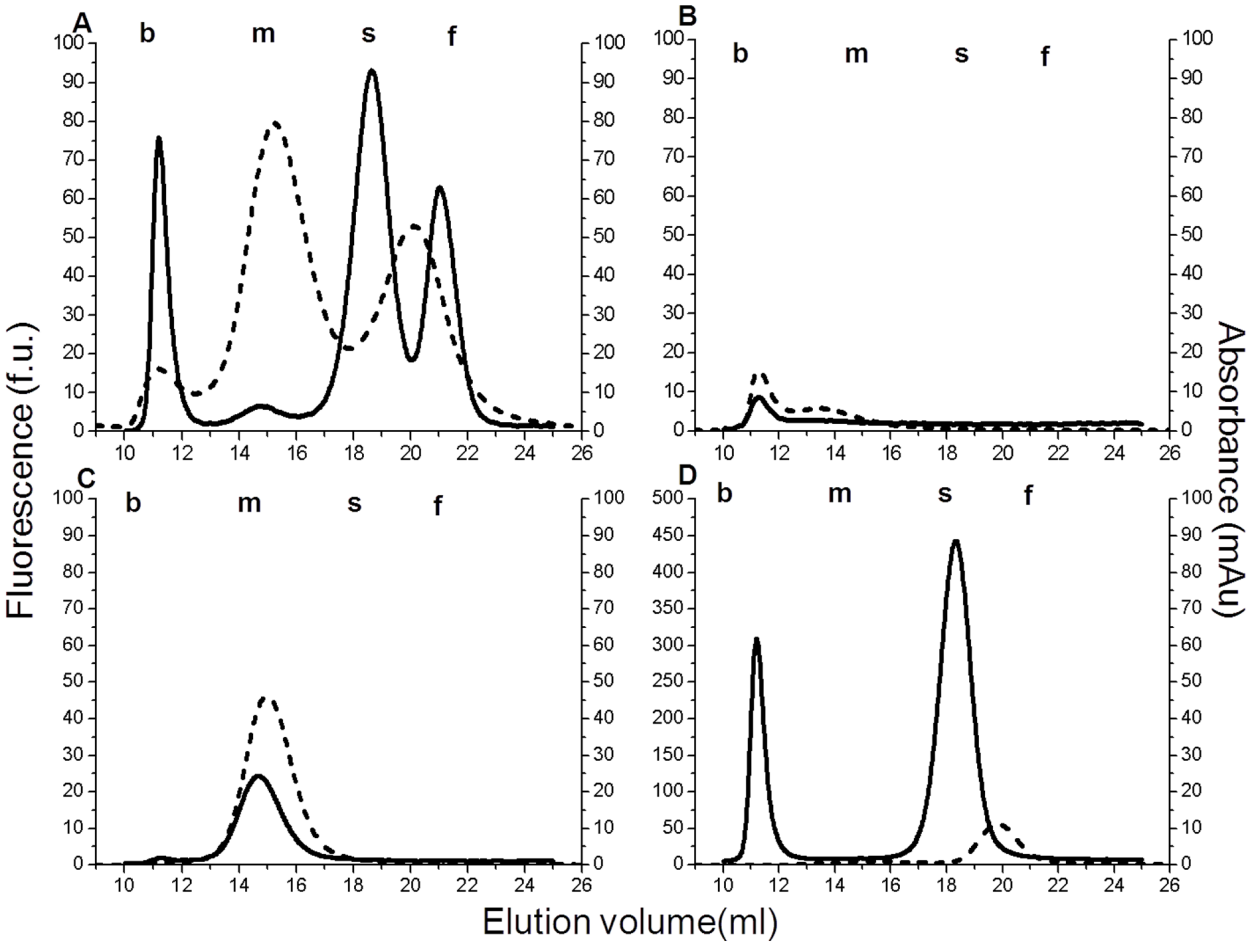
GGT-specific (continuous line) and total cholesterol (dashed line) elution profiles in a plasma pool (pool-1) representative of four experiments, and in the lipoprotein fractions isolated from it. A) Total plasma; B) VLDL (elution volume: 11.3 ml; density 0.950–1.006 g/ml; Stokes radius: 30–80 nm); C) LDL (15.0 ml; 1.006–1.063 g/ml; 18–25 nm); D) HDL (20.0 ml; 1.063–1.21 g/ml; 8–11 nm). Lipoproteins were separated by ultracentrifugation on a NaBr discontinuous density gradient. Elution profiles were obtained by size-exclusion chromatography associated with a post-column reaction specific for GGT or total cholesterol.

The comparison between the elution profile of GGT activity and total cholesterol, performed on the same plasma pool sample (**Fig. 1A**), showed that b-GGT and m-GGT fractions eluted with VLDL (elution volume: 11.3 ml; Stokes radius: 30–80 nm) and LDL (15.0 ml; 18–25 nm) cholesterol peaks, respectively. The remaining s-GGT and f-GGT fractions (elution volume 19.0 ml and 21.0 ml, respectively) presented with an elution profile partially overlapping the HDL (20 ml; 8–11 nm) cholesterol peak.

On these bases, VLDL lipoprotein (density 0.950–1.006 g/ml), LDL (density 1.006–1.063 g/ml) and HDL (density 1.063–1.21 g/ml) were separated by discontinuous density-gradient ultracentrifugation and then analysed to evaluate the associated GGT fractions. Interestingly, b-GGT fraction was mainly recovered with HDL lipoprotein (**Fig. 1D**) and in minor quantity with VLDL lipoprotein (**Fig. 1B**); m-GGT and s-GGT fractions were observed only in LDL and HDL samples, respectively (**Fig. 1C, 1D**); on the contrary, f-GGT fraction was not associated with any density-gradient lipoprotein fraction, being detectable only in the lipoprotein-free plasma sample (data not shown). The differential distribution of GGT fractions according to density was confirmed in four independent experiments.

### Plasma GGT fractions: sensitivity to papain and deoxycholic acid treatment

**Figure 2:**
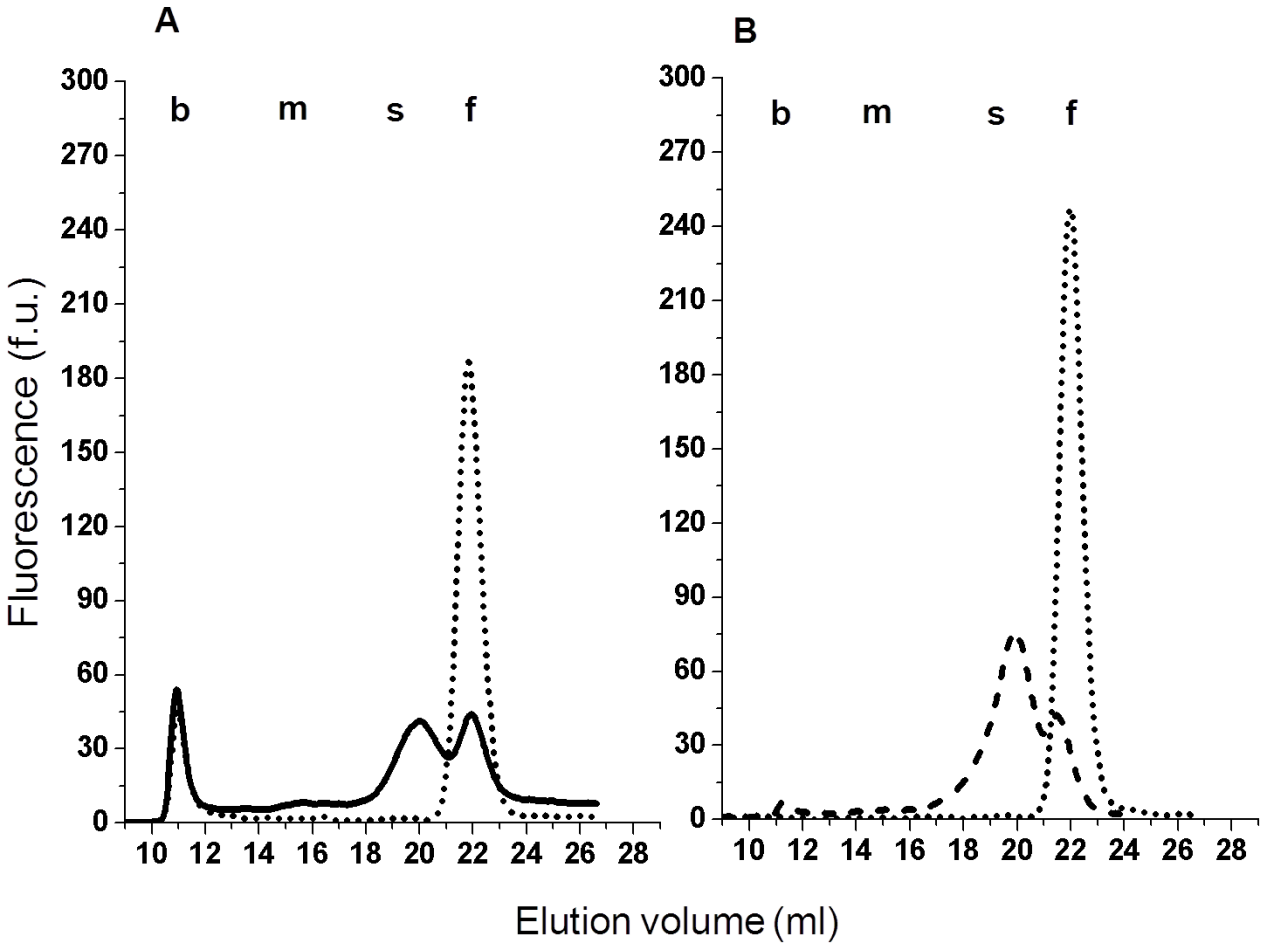
Papain and DOC effects on plasma samples. Effect of papain (25 mmol/l, 24 h, RT) and DOC (1% w/v, 1 h, 37°C) treatments on GGT fractions in a plasma sample representative of four independent experiments. A) GGT-specific elution profile in the untreated control [continuous line; GGT activity (U/L): total = 38.8; b-GGT = 8.9; m-GGT = 3.1; s-GGT = 14.1; f-GGT = 12.6] and after papain treatment [dotted line; GGT activity (U/L): total = 49.8; b-GGT = 7.5; m-GGT = n.d.; s-GGT = n.d.; f-GGT = 42.3]. B) GGT-specific elution profile after DOC treatment [dashed line; GGT activity (U/L): total = 45.0; b-GGT = 1.3; m-GGT = 2.4; s-GGT = 31.2; f-GGT = 10.1] and after DOC and papain treatments [dotted line; GGT activity (U/L): total = 57.0; b-GGT = n.d.; m-GGT = n.d.; s-GGT = n.d.; f-GGT = 57.0]. n.d.: not detectable.

In order to better characterize the GGT protein associated with the four plasma fractions, the presence of the N-terminal, membrane anchoring peptide of GGT heavy chain was evaluated. Plasma samples were treated with papain, a cysteine protease known to promote the release of a soluble, active enzymatic form of GGT from membranes [25]. Actually, papain treatment did not affect b-GGT fraction properties, *i.e.* activity, dimension and gel filtration elution volume. On the contrary, both m-GGT and s-GGT fractions were no longer detectable after papain treatment, all GGT activity being recovered in the f-GGT fraction (**Fig. 2A**). Such post-papain f-GGT peak presented with the same elution volume of untreated plasma f-GGT, but its area was equal to the sum of m-, s- and f-GGT peaks detectable in the corresponding untreated plasma.

As regard detergents sensitivity, the treatment of plasma samples with DOC – an anionic detergent physiologically present in bile – resulted in the reduction of b-GGT peak with a corresponding increase of s-GGT fraction, the latter presenting with the same elution volume of s-GGT in the untreated plasma. On the contrary, m-GGT and f-GGT fractions were not altered by DOC treatment (**Fig. 2B**). Interestingly, when also papain was added to DOC treated samples all enzymatic activity was recovered in the f-GGT fraction, (**Fig. 2B**). Sensitivity to papain and deoxycholic acid of plasma GGT fractions was confirmed in four independent experiments.

### Bile GGT fractions: sensitivity to papain and deoxycholic acid treatment

**Figure 3:**
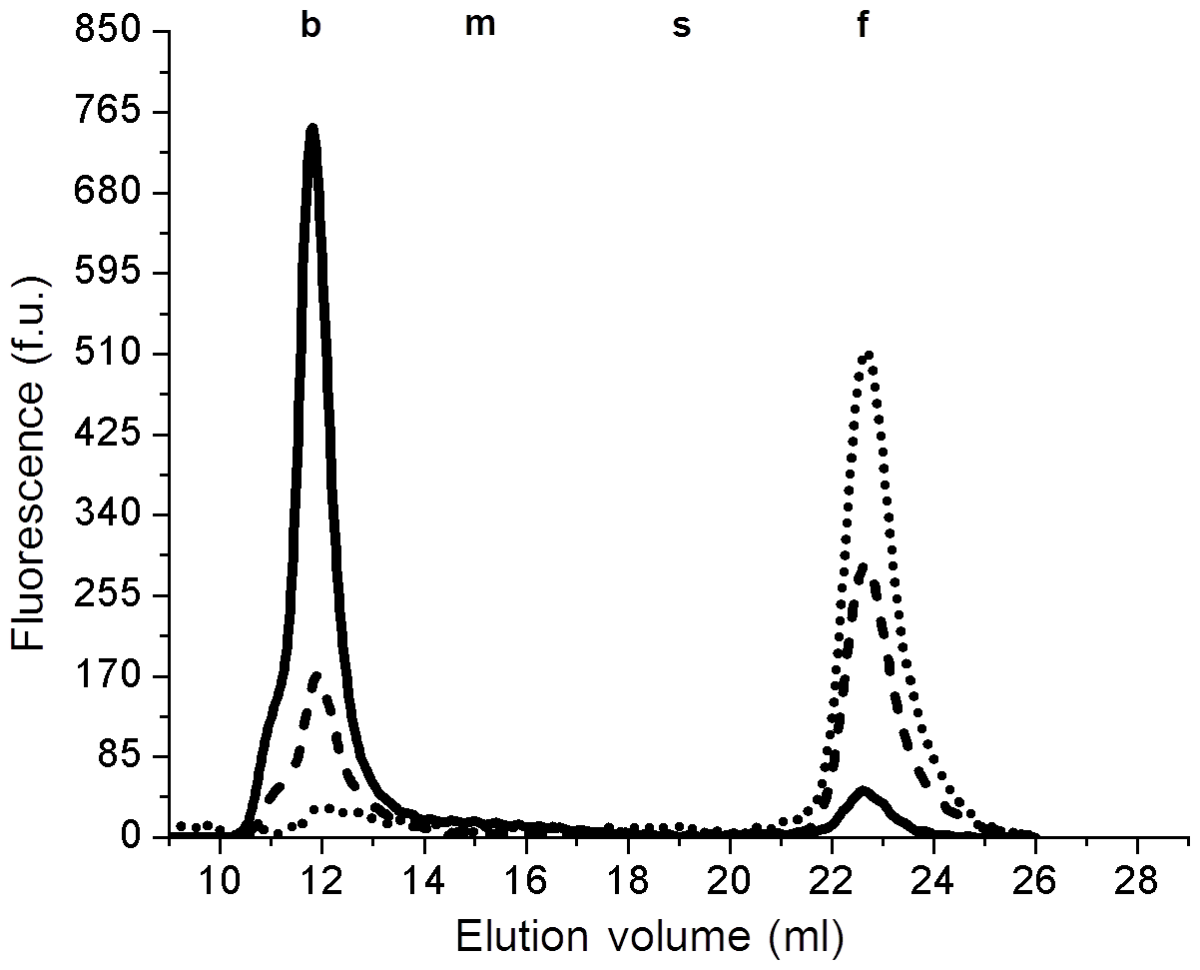
Papain and DOC effects on bile samples. Effect of papain (25 mmol/l, 24 h, RT) and DOC (1% w/v, 1 h, 37°C) treatments on GGT fractions in sample of human bile representative of four independent experiments. Untreated control [continuous line; GGT activity (U/L): total = 127.5; b-GGT = 106.2; f-GGT = 21.3], after papain treatment [dashed line; GGT activity (U/L): total = 171.22; b-GGT = 62.1; f-GGT = 109.1] and after DOC and papain treatments [dotted line; GGT activity (U/L): total = 217.6; b-GGT = 19.6; f-GGT = 198.0].

Human bile GGT activity is higher than plasma GGT values (median: 112.5 U/L, 25°–75° percentile: 73.9–183.2 U/L, min-max 55.8–218.8 U/L, n = 7). In all examined samples, about 90% of bile GGT activity was found to be associated with a high MW fraction corresponding to plasma b-GGT (**Fig. 3**), while the remaining part corresponded to plasma f-GGT fraction. Notably, bile b-GGT peak showed a left side asymmetry, possibly suggesting a heterogeneous composition of such fraction, due to the presence of at least two distinct GGT-bearing molecular complexes: the first, with a higher elution volume/lower molecular mass, corresponding to plasma b-GGT complex, and the other, with a lower elution volume/higher molecular mass, detectable only in bile samples. Treatment of bile with papain resulted in a partial reduction of b-GGT peak and a corresponding increase of f-GGT peak (**Fig. 3**). Similarly to plasma, the combined treatment with DOC and papain allowed the complete recovery of b-GGT activity into f-GGT fraction (**Fig. 3**). Both papain and DOC plus papain treatments resulted in an increase of total GGT activity in bile samples (data not shown). Sensitivity to papain and deoxycholic acid of bile GGT fractions was confirmed in four independent experiments.

### Microparticles and exosomes recovery from plasma and bile

**Figure 4:**
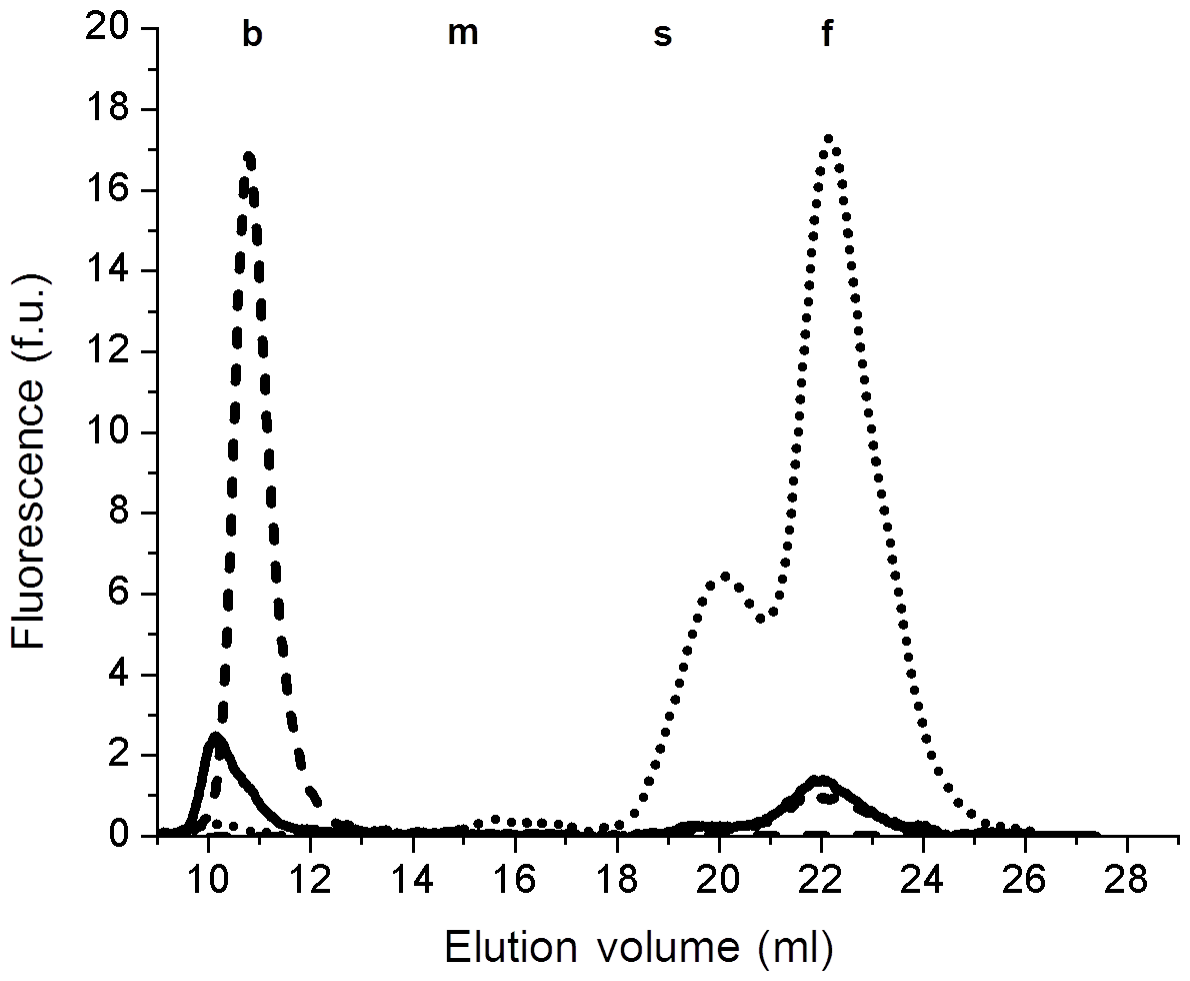
GGT activity of isolated microparticles and exosomes. GGT-specific elution profiles of isolated microparticles (continuous line), exosomes (dashed line) and microparticles/exosomes-free plasma (dotted line) samples. Microparticles were obtained from plasma by centrifugation (10,000 g, 45 min, 4°C) whereas exosomes were recovered by ultracentrifugation (100,000 g, 2 h, 4°C) of the corresponding microvesicles-free supernatant. Representative chromatograms of five independent experiments are reported.

When samples of isolated plasma microparticles were analysed by gel filtration chromatography, a GGT activity peak with an elution volume (10.1 ml) lower than that of plasma b-GGT fraction (10.9 ml) was detected (**Fig. 4**). On the other hand, when samples of isolated plasma exosomes were analysed, we observed a GGT activity peak with the same elution volume of plasma b-GGT fraction (10.9 ml). This latter peak was absent in the corresponding ultracentrifuged exosome-free plasma supernatants, where only m-, s- and f-GGT fractions were detectable (**Fig. 4**).

As regard bile samples, all b-GGT activity was recovered in the pellet containing exosomes, while f-GGT remained in supernatant (data not shown). These findings were confirmed in five independent plasma samples and four independent bile samples.

### Electron microscopy of exosomes purified from plasma and bile

**Figure 5:**
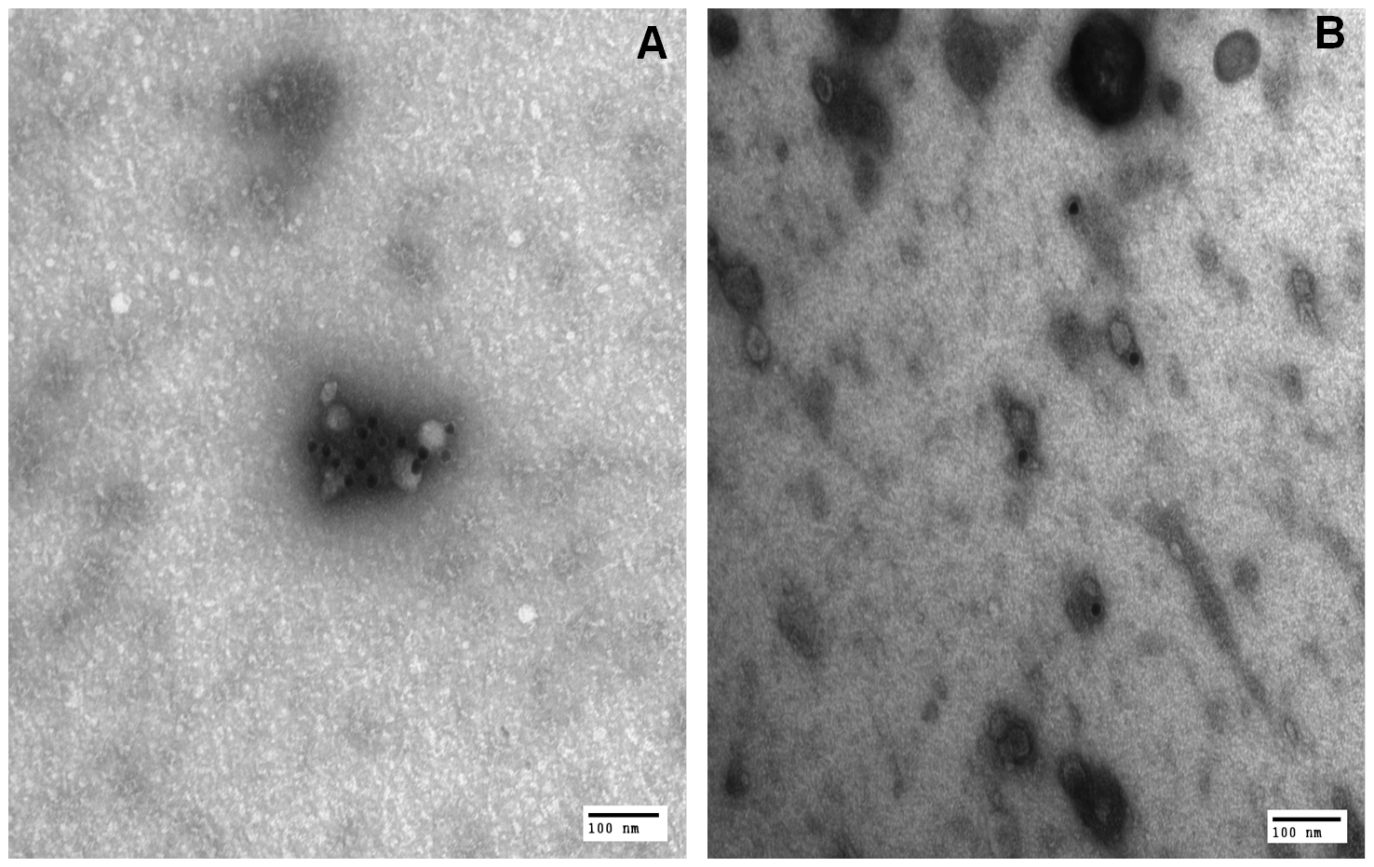
GGT localization on exosomes by transmission electron microscopy. Exosomes from A) plasma and B) bile were incubated with a primary antibody directed against GGT and with a secondary 15 nm gold particle-conjugated antibody. After fixation with glutaraldehyde, samples were negatively stained with uranyl acetate and examined under a transmission electron microscope.

Electron microscopy analysis of plasma and bile exosomes allowed us to confirm the presence of membrane microvesicles with the typical size of exosomes (20–50 nm). The presence of GGT protein on part of such microvesicles was confirmed by immunogold staining (**Fig. 5**).

## Discussion

The main finding of this study is the first characterization of fractional GGT, which revealed an unexpected complexity. According to early studies [26], both gel filtration chromatography and ultracentrifugation procedures revealed a fair analogy between high molecular weight GGT fractions (b-, m- and s-GGT) and circulating lipoprotein VLDL, LDL and HDL. Nevertheless, our results demonstrated that none of the three GGT fractions could be completely identified with the corresponding class of lipoprotein sharing the same MW. Indeed, if we exclude s-GGT, all the other GGT fractions have distinctive features as compared to lipoproteins, and they can be physically separated from them. These results are against what suggested in early studies, *i.e.* that circulating lipoproteins are GGT carriers and that GGT is too lipophilic to circulate in a free form [26]. Furthermore, we also observed that plasma f-GGT fraction has a MW corresponding to the free GGT protein, thus demonstrating that part of plasma GGT do not require any carrier, such as lipoproteins [26] or albumin [27], to be transported in blood.

Our results showed that plasma b-GGT fraction is constituted of membrane microvesicles (microparticles and exosomes), b-GGT dimension and density (30–80 nm; 1.06–1.21 g/ml) being compatible with those of membrane microvesicles (40–100 nm; 1.15–1.27 g/ml) [28]. According to this interpretation, b-GGT was also shown to be sensitive to DOC action, i.e. a detergent that can disrupt membrane microvesicles [29], and immunogold analysis confirmed the association between GGT protein and exosomes.

It was demonstrated that cholesterol-rich micro domains of the plasma membrane, the so called “lipid rafts”, play a crucial role in processes of exo/endocytosis and in the biogenesis of microvesicles, and that secreted microvesicles possess high levels of cholesterol rafts [28], [29]. Interestingly, plasma membrane GGT was found to be specifically localized in lipid rafts, where it is associated with tetraspanin CD81 [30], *i.e.* a marker that is usually detectable also in exosomes and microparticles [31]. This particular localization of GGT may thus help to explain why such enzyme is released in the extracellular compartment in association with microvesicles.

More complicated seems to be the biogenesis of the other GGT fractions (m-GGT, s-GGT and f-GGT) that might arise from progressive modifications of the b-GGT fraction. In this perspective our results demonstrated that b-GGT is sensitive to protease papain only after a pre-treatment with DOC, this possibly suggesting a protective role of b-GGT phospholipid bilayer on papain cleavage site. On the other hand, the fact that DOC is able to convert b-GGT fraction into micelles of s-GGT size suggests that these latter might be constituted of micelles of bile acids, and that this conversion would take place in the extracellular compartment, after b-GGT secretion. Similarly to s-GGT, also m-GGT fraction appeared to be sensitive to papain but insensitive to DOC: these characteristics, together with its physical properties (size, density), suggest that m-GGT could be constituted of micelles of bile acids with sizes greater than s-GGT ones. Finally, f-GGT corresponds to the free, soluble form of the enzyme, lacking the N-terminal anchoring peptide, which is present in all other fractions. Our results indicate that it could originate directly from both m-GGT and s-GGT fraction as a consequence of a proteolytic cleavage.

In this perspective, the analysis of human bile, rather than solving part of the problem, raised other questions about fractions composition. Basing on the assumption that both m-GGT and s-GGT fractions are formed by micelles of bile acids and GGT, we also compared the human bile fractional profile with that of human plasma treated with DOC. Unexpectedly, the elution profile of bile GGT showed the presence of only two fractions, corresponding to plasma b-GGT and f-GGT. Actually, the lack of m-GGT and s-GGT fractions may be due to the low bile acids concentration in hepatic bile, which is about 10 times lower than the amount of DOC able to convert plasma b-GGT into s-GGT (1.7 mmol/l *vs.* 24 mmol/l). Further studies will have to focus on fractional GGT in gallbladder bile, where bile acids concentration is 5–10 times higher than that of hepatic bile. Regarding the two bile fractions, they revealed a greater heterogeneity as compared to plasma ones. Again f-GGT represents the free enzyme, nevertheless our data indicate that b-GGT fraction may be composed of two distinct molecular complexes: the first with exosomes size and properties, and the other with a size similar to that of exosomes but sensitive to papain action. Interestingly, about 80% of biliary GGT activity can be recovered in the fraction presenting a density of 1.123 g/ml, the richest content of proteins (55% of total) and bile acids (35%), but showing the lowest amount of phospholipids (17%) and, even, undetectable levels of cholesterol [32]. This composition seems to suggest that this papain-sensitive part of bile b-GGT could be composed of bile acid micelles.

Another specific property of fractional bile GGT is that following papain treatment – either in the presence or in the absence of DOC – the total amount of GGT activity recovered in the resulting f-GGT fraction was higher than expected. It can be envisaged that the molecular context surrounding biliary GGT may alter not only its sensitivity to proteases, but also the kinetic of the enzyme activity, *e.g.* by influencing the accessibility of the GGT substrate to the active site of the enzyme [33].

Against this background, it is clear that a better knowledge of physical and molecular properties of fractional plasma GGT would help both to understand their role in the pathogenesis of diseases, and to improve their clinical utilization as biomarkers of disease. *In vitro* studies demonstrated that different cell types release b-GGT in culture medium [34] as well as upon activation [35]. It can be hypothesized that also *in vivo* several GGT expressing tissues may contribute to b-GGT in plasma and in other biological fluids. Proteomic analysis revealed that microvesicles have a unique protein composition that varies depending on cellular origin [36]: the identification of cell specific markers in the b-GGT fraction would thus allow the identification of major tissues contributing to plasma GGT in all the pathological conditions associated with its elevation.

Fractional GGT analysis of plasma obtained from healthy subjects, patients with non-alcoholic fatty liver disease, viral or alcoholic chronic hepatitis showed the presence of the same four GGT fractions but with different amount of associated GGT activity [18]–[20]. This suggests that different disease status might influence the availability of carriers but not their interaction with the enzyme. Similarly, we showed in this study that bile samples were characterized by the presence of the same two GGT fractions (b-GGT and f-GGT) indicating a shared mechanism of release.

Our results allowed us to have a first look inside the nature of fractional GGT, but further studies will be required to fully characterize their exact composition and the compartments in which they are generated.
